# Teaching signal detection theory with pseudoscience

**DOI:** 10.3389/fpsyg.2015.00762

**Published:** 2015-06-03

**Authors:** Nicole D. Anderson

**Affiliations:** Department of Psychology, MacEwan UniversityEdmonton, AB, Canada

**Keywords:** signal detection, pseudoscience, pedagogy, decision-making, scientific thinking

## The utility of signal detection theory

Signal detection theory (SDT) is a technique that can be used to evaluate sensitivity in decision-making. Initially developed by radar researchers in the early 1950s (Peterson et al., 1954), the value of SDT was quickly recognized by cognitive scientists and adapted for application in human decision-making (Tanner and Swets, 1954; Green and Swets, 1966). The general premise of SDT is that decisions are made against a background of uncertainty, and the goal of the decision-maker is to tease out the decision signal from background noise. SDT can be applied to any binary decision-making situation where the response of the decision maker can be compared to the actual presence or absence of the target. The advantage of SDT as a measure of decision-making is that it provides a unitless measure of sensitivity, regardless of subject bias, that can be compared to other sensitivities over widely different situations.

SDT has been applied within a broad range of topics, including memory research (e.g., Banks, 1970), accuracy in radiology diagnostics (e.g., Obuchowski, 2003), and sustained attention in individuals with ADHD (e.g., Huang-Pollock et al., 2012). Further testament to the utility of SDT comes from the fact that SDT is often discussed in introductory courses and textbooks (e.g., Wade et al., 2013; Lilienfeld et al., 2015). However even with the ubiquity of SDT as an evaluative tool, the mechanics of SDT are not typically discussed in undergraduate textbooks, suggesting that many students are not taught how to practically apply SDT in their university careers. One reason for this is that the procedure itself may appear deceptively complex. Most SDT sources discuss the theory with a rigor that is beyond the mathematical knowledge of most undergraduate students (Fisher, 2014). However, the application of basic SDT principles requires only rudimentary statistical knowledge and can easily be taught at an undergraduate level.

Another reason that SDT may not typically be covered in the undergraduate curriculum is a lack of compelling examples to demonstrate the utility of SDT. Oftentimes, examples are related to sensory performance and the practical application of SDT techniques to more high-level decision-making situations is not immediately apparent to students. For example, Goldstein (2014) and Wolfe et al. (2015), two common introductory textbooks for Sensation and Perception, discuss the theory of SDT in relation to hearing sensitivity in the context of noise. However, there are more inherently interesting examples that can be used in the classroom to demonstrate the versatility of SDT to learners. Given the importance of active learning through concrete examples when learning inherently abstract statistical principles such as SDT (e.g., Watts, 1991; Garfield and Ben-Zvi, 2007) the onus is on educators to develop compelling examples to capture the interest of undergraduate students. Here, I argue that pseudoscientific “principles” can be used to demonstrate the power of SDT, given that many pseudoscientific results can be explained as the detection of patterns in meaningless noise.

## The mechanics of signal detection theory: a brief overview

**Figure 1:**
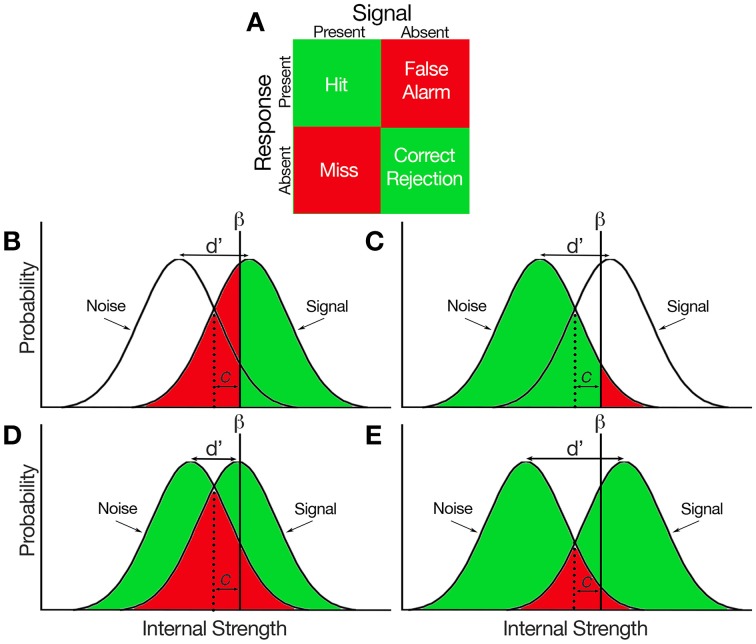
**Hypothetical representations of the signal and noise probabilities distributed within a decision-maker**. **(A)** Response matrix of all signal-response combinations that can be made in a binary decision task. Green indicates correct decision, red indicates incorrect decision. **(B)** Proportions of hits and misses represented under the signal distribution. β reflects the subject criterion, *c* reflects bias, and d' reflects sensitivity which represents the difference in position between the two distributions. **(C)** Proportions of false alarms and correct rejections represented under the noise distributions. **(D)** A condition which hypothetically reflects low subject sensitivity. When the distributions are closer together (i.e., d' is smaller), the difference between the proportion of hits and false alarms is lower. **(E)** A condition which reflects high subject sensitivity. When the distributions are farther apart (i.e., d' is larger), the difference between the proportion of hits and false alarms is higher.

The basic premise behind SDT is that both signal and noise are represented probabilistically within the decision-maker, and the extent to which those representations overlap can be estimated based on the decision-maker's responses and whether or not the signal is present (Figure 1A). The decision-maker bases their decision relative to their criterion (β), where a signal will be reported present when the internal signal is stronger than β and absent when the internal signal is weaker than β. A hit represents the probability that the subject reports the signal present when it is (Figure 1B, green) and a false alarm represents the probability that the subject reports the signal present when it is absent (Figure 1C, red). Alternatively, a miss represents the probability that the subject reports the signal absent when it is present (Figure 1B, red) and a correct rejection represents the probability that the subject reports the signal absent when it is absent (Figure 1C, green). All response probabilities are reflected as a part of the area underneath a normal curve. If the probability of each response type is therefore known, both the signal and the noise distributions can be estimated based on simple statistical principles.

Determining the z-score (i.e., the standard deviation) of the probabilities associated with each distribution will provide an estimate of the properties of the underlying distributions. The *z*-value associated with the probability of a hit (*P*_hit_) will reflect where β is positioned relative to the signal distribution. Similarly, the *z*-value associated with the probability of a false alarm (*P*_FA_) will reflect the position of β relative to the noise distribution. The difference in the position of β (or the difference in z-scores) therefore reflects the difference in position between the signal and noise distributions. Thus, sensitivity can simply be estimated as:

$$d^{\prime }=Z\left(P_{hit}\right)-Z\left(P_{FA}\right)$$

which means that sensitivity reflects both the probability of a hit and the probability of a false alarm. A small d' (i.e., *P*_hit_ is close in value to *P*_FA_) would reflect a condition where the signal and noise distributions share a substantial amount of overlap (Figure 1D). On the other hand, a large d' (i.e., *P*_hit_ is considerably greater than *P*_FA_) would occur when both distributions shared very little overlap (Figure 1E). Note that d' is independent of where β is placed, thus d' is a measure of performance that is independent of subject bias.

The bias of the observer can also be estimated from the probabilities of hits and false alarm rates. Bias can be reported as the difference between the placement of β by the observer and the placement of β by an unbiased observer (i.e., an ideal observer) who would demonstrate equal proportions of misses and false alarms. This value, typically referred to as *c*, can be estimated as the average of the z-scores for both *P*_hit_ and *P*_FA_, thus:

$$c\;=\;-\frac{Z\left(P_{hit}\right)+\;Z\left(P_{FA}\right)}{2}$$

*c* is negative when β is placed further left along the distribution (i.e., a liberal criterion), meaning the subject is more likely to report the signal is present. On the other hand, *c* is positive when β is placed further right (i.e., a conservative criterion), meaning the subject is less likely to report the signal is present. The absolute value of *c* provides an indication of the strength of the subject bias. This discussion is only meant as a brief review of SDT; for a more comprehensive summary of the principles of SDT, see Stanislaw and Todorov (1999) or MacMillan and Creelman (1991).

## Pseudoscience as a tool for teaching signal detection theory

Many pseudosciences present excellent examples that can be used to demonstrate the value of SDT to learners. While believers of pseudoscientific principles claim to be sensitive to those principles, sensitivity in these situations is not typically considered with respect to false alarms. For example, the efficacy of homeopathic treatments is typically considered as a placebo effect, which can be understood within the SDT framework (Allan and Siegel, 2002). If an individual claimed that a particular homeopathic treatment was effective (hit), but would also be likely to claim that a placebo was effective (false alarm), the associated d' for that individual would be low. The value of SDT in this situation is that it provides an objective measure of an individual's sensitivity outside of subject bias.

There are several advantages to using pseudoscience to teach SDT. First, pseudoscience is an excellent vehicle for teaching scientific thinking (Schmaltz and Lilienfeld, 2014), and students find the discussion of pseudoscientific concepts to be inherently interesting. Astrology, homeopathy, psychic abilities, quantum mysticism, and paredolia are all pseudoscientific topics that can provide compelling examples of how performance can be objectively measured using SDT. Second, response differences between believers and skeptics tend to be reflected in the bias of the responses rather than a difference in sensitivity. For example, research has demonstrated that paranormal believers are more likely to detect patterns in noise than are skeptics; however, this increase in detection only reflects a more liberal bias and not an increase in d' (e.g., Riekki et al., 2013; Van Elk, 2013). Pseudoscientific examples present excellent demonstrations of how changes in β would be reflected in the responses of believers vs. skeptics. Finally, thinking about pseudoscience in the context of SDT forces students to critically think about what subject responses reflect, and whether or not those responses are an objective measure of behavior. In other words, just because an individual claims to detect a signal does not mean that they do, and it is important to consider this when designing a scientific study.

Here, I present two examples that can be used as to demonstrate the value of SDT using pseudoscientific principles. These examples can be easily adapted to utilize many pseudoscientific principles that may be taught in undergraduate psychology classes. The only statistical knowledge that is required on behalf of the student is a basic understanding of z-scores and the normal distribution. An alternative exercise for students is to formulate their own research designs that would allow them to investigate pseudoscientific principles using SDT with the following examples as a framework. Either approach would provide students with valuable hands-on experience for using SDT to objectively assess human decision-making.

## Examples

### Example 1: paredolia

Electronic voice phenomena (EVP) is a claim by parapsychologists that spirit voices can be detected in random radio noise. Dr. James conducts an experiment with a spiritualist and a skeptic to determine if EVP can be reliably detected by the spiritualist. Each subject is presented with 100 five-second sound clips, where 50 of the sound clips contain a very weak voice signal and the other 50 clips contain random noise. In each trial, the subjects report whether the sound clip contained a voice. In the voice condition, the spiritualist detected the presence of the voice 92% of the time, whereas the skeptic detected the voice 58% of the time. In the noise condition, the spiritualist detected the presence of the voice 48% of the time, whereas the skeptic detected the voice 9% of the time. Did Dr. James find that the spiritualist was more sensitive than the skeptic?

#### Solution

The proportion of hits made by the spiritualist is 0.92 (Z_hit_ = 1.4051) whereas the proportion of false alarms is 0.48 (Z_FA_ = −0.0502). Thus the sensitivity of the spiritualist is:

$$d^{\prime }=\left(1.4051\right)-(-0.0502)=1.4553$$

and the bias of the spiritualist is:

$$c=-\frac{\left(1.4051\right)+\left(-0.0502\right)}{2}=-0.6774$$

The proportion of hits by the skeptic is 0.58 (Z_hit_ = 0.2019) and the proportion of false alarms is 0.09 (Z_FA_ = −1.3408), thus the sensitivity of the skeptic is:

$$d^{\prime }=\left(0.2019\right)-(-1.3408)=\;1.5427$$

and the bias of the skeptic is:

$$c=-\frac{\left(0.2019\right)+\left(-1.3408\right)}{2}\;=\;0.5695$$

Therefore, the spiritualist was not more sensitive to EVP than the skeptics, but did demonstrate a more liberal bias than the skeptic, who demonstrated a more conservative bias.

### Example 2: psychics

A psychic detective is consulted by the Metro city police department for multiple missing person cases. Of these cases, 50 bodies are discovered. Before discovery, the psychic claimed that 31 of the bodies would be discovered close to water. After the bodies were discovered, it was determined that 17 of the bodies the psychic predicted would be close to water were within 500 m of a body of water. On the other hand, 11 of the bodies that the psychic did *not* claim were close to water were also within 500 m of a body of water. Was the psychic sensitive to the location of the bodies?

#### Solution

The proportion of bodies the psychic correctly determined were close to water was 0.6071 (17/28) whereas the proportion that the psychic claimed were close to water but were not was 0.6364 (14/22). The associated z-scores for the hits and false alarms are therefore Z_hit_ = 0.2718 and Z_*FA*_ = 0.3489. Therefore the sensitivity of the psychic is:

$$d^{\prime }=\left(0.2718\right)-\left(0.3489\right)=-0.0771$$

and the bias is:

$$c=\;-\frac{\left(0.2718\right)+\left(0.3489\right)}{2}\;=\;-0.3104$$

Thus, the psychic was not sensitive in predicting the location of the bodies, but did demonstrate a liberal bias for reporting that bodies would be discovered close to water.

### Conflict of interest statement

The author declares that the research was conducted in the absence of any commercial or financial relationships that could be construed as a potential conflict of interest.
